# A national survey of Intensive Care Medicine Services in Portugal: where we are and the road ahead

**DOI:** 10.62675/2965-2774.20250302

**Published:** 2025-01-30

**Authors:** José-Artur Osório de Carvalho Paiva, Rui Alberto Lomelino Araújo, Paulo Jorge Coimbra Martins, António Jose Pereira Pais-Martins, Fernando Manuel Ferreira Araújo

**Affiliations:** 1 Unidade Local de Saúde de São João Intensive Care Service Porto Portugal Intensive Care Service, Unidade Local de Saúde de São João, EPE - Porto, Portugal.; 2 University of Porto Faculty of Medicine Porto Portugal Faculty of Medicine, University of Porto - Porto, Portugal.; 3 Unidade Local de Saúde de Matosinhos Intensive Care Service Porto Portugal Intensive Care Service, Unidade Local de Saúde de Matosinhos, EPE - Porto, Portugal.; 4 Unidade Local de Saúde de Coimbra Intensive Care Service Coimbra Portugal Intensive Care Service, Unidade Local de Saúde de Coimbra, EPE - Coimbra, Portugal.; 5 University of Coimbra Faculty of Medicine Coimbra Portugal Faculty of Medicine, University of Coimbra - Coimbra, Portugal.; 6 Unidade Local de Saúde de Lisboa Ocidental Intensive Care Service Lisboa Portugal Intensive Care Service, Unidade Local de Saúde de Lisboa Ocidental, EPE - Lisboa, Portugal.; 7 Unidade Local de Saúde de São João Imunohemotherapy Service Porto Portugal Imunohemotherapy Service, Unidade Local de Saúde de São João, EPE - Porto, Portugal.

**Keywords:** Intensive care medicine, Critical care, Health services, Organizations, Hospitals, Goals, Beds, Reference network, Referral and consultation, Nursing staff, Surveys and questionnaires, Portugal

## Abstract

**Objective:**

The goal of this study was to assess the Portuguese Intensive Care Referral Network, namely the mission and organization of the Portuguese National Health Service Intensive Care Medicine Services and patient flows between them.

**Methods:**

The study was based on the responses to a semi-structured questionnaire by the directors of the forty-one Intensive Care Medicine Services, characterizing four domains: a) number, type, and management of beds; b) human resources and their consumption; c) outreach, including activities in the resuscitation room, intra-hospital emergency team and follow-up clinics; and d) referral network.

**Results:**

The number of active Intensive Care Medicine Services beds in Portugal markedly increased in the last 12 years, but the beds/habitant ratio is still below the Organization for Economic Cooperation and Development average. The activation of all installed beds would likely allow for the reduction of the hospital care gap perceived by many of the Intensive Care Medicine Services directors. There is significant geographic heterogeneity in the beds/habitant ratio and in the performance of outreach activities. The number of intensivists is rapidly growing, but nursing staff should be augmented, especially rehabilitation nurses. The referral network is globally complied, but the secondary transport of critical patients needs improvement and an electronic information system, which can be constantly updated, is seen as a relevant decision aid.

**Conclusion:**

Although intensive care medicine has significantly strengthened in the last 12 years, both in number of beds and in role and mission, there is still relevant heterogeneity in the beds/habitant ratio and in the performance of outreach activities among different Intensive Care Medicine Services.

## INTRODUCTION

Modern intensive care medicine (ICM) began with the development of the iron lung and of negative pressure ventilation used for the polio epidemic in the early fifties of the last century. Dr. Bjørn Ibsen organized the first European intensive care unit (ICU) to provide respiratory care for polio patients in the Blegham Hospital in Copenhagen.^(1)^ A few years later, in 1958, Max Harry Weil and Hebert Shubin opened a four-bed shock ward in LA County-USC Medical Center, Los Angeles and Peter Safar opened a multidisciplinary ICU at Baltimore City Hospital.^(2)^

Since those early days, huge improvements have been made in terms of monitoring and organ support technology, process of care, and understanding of the pathophysiology of the diseases and syndromes that affect critically ill patients. The ability to support temporarily and, in some cases, to replace the function of multiple-organ systems in the face of critical illness and injury is the core capability that underpins ICM. ICU technology facilitates prompt bedside diagnosis, monitoring, and interventions in settings of multiorgan failure, allowing real-time modulation of treatment and time for treatment to work.

Modern ICM is one of the fastest-growing hospital specialties in terms of volume of cases, staff, and institutional role and mission. In the last decades, there was a decrease in the total number of hospital beds, but the number of ICU beds increased, both in Europe and in the United States.^(3-5)^ As the need for intensive care continues to increase, the ratio of ICU beds to hospital beds will continue to rise as ICM serves a larger and increasingly important role at the center of acute hospital care.^(6)^

Moreover, ICM was transformed into an organized system of care for the critically ill, whatever the location, that ensures delivery of timely and expert treatment to patients, extending this provision to all hospital and even pre-hospital locations and becoming a continuum of care.^(7)^ Intensive care teams operate in the resuscitation room of the emergency department, in intra-hospital medical emergency teams and in follow-up clinics, perceiving that outcome is very much determined by pre-ICU time and care and that long-term outcomes are important and affected by pre-ICU and ICU variables.^(8)^

In Portugal, the first ICU was introduced in 1959. In the 1980s, several ICUs were organized as services, and the first regulated process for formal certification as an intensivist was implemented. In the last 25 years, several Intensive Care Medicine Services (ICMS) expanded their activity beyond the walls of the ICU and the process of outreach developed towards the emergency department, through intrahospital emergency teams, and into follow-up clinics. In 2015, ICM was instituted as a primary specialty, and its residency was legislated in 2016. In the same year, the first Intensive Care Referral Network was approved and published, with a four-year (2016-2020) development plan.^(9)^ This document was revised in 2020, during the first wave of the COVID-19 pandemic.^(10)^ However, Portugal remained as one of the countries in Europe with fewer intensive care beds per 100,000 residents until the COVID-19 challenge led to a development plan aimed at a sustained increase in the number of these beds. However, not only has an assessment of the impact of this development plan not yet been published, but the mission of ICMS is also not clearly defined, either by regulation or by law, and significant heterogeneity exists throughout the country, as is in fact the case throughout Europe and the world.

The goal of this study was to assess the Portuguese Intensive Care Referral Network, namely the mission and organization of the Portuguese National Health Service ICMS and patient flows between them.

## METHODS

The study was performed by a working group nominated by the Portuguese National Health Service (PT-NHS) executive board for the revision of the Portuguese Intensive Care Referral Network. The group included four experienced intensivists with long clinical and managerial experience in ICM.

A semi-structured questionnaire was prepared by the working group and sent to the directors of the forty-one ICMS of the PT-NHS. The fifty-nine questions were intended to characterize the current situation of ICM in four domains: a) number and type of beds and their management; b) human resources and their consumption; c) outreach, including activities in the resuscitation room, in the intra-hospital emergency team, and in follow-up clinics; d) referral network.

The answers were given through a link and a period of two weeks, in April 2024, was allocated for the purpose. After that period, the link was closed and submission or re-submission was impossible. During that period, none of the responders could view the answers of the other respondents, and only the coordinator of the working group could view all the answers. The answers were then analyzed by the four elements of the working group.

For longitudinal comparison, data regarding the numbers of ICMS beds in 2010, 2019, and 2022 and regarding outreach activities in 2022 were obtained. Data regarding the Portuguese population and its distribution were collected from the "Statistics Portugal" web portal,^(11)^ taking into account the health referral areas of the hospitals of the five Portuguese health regions and Madeira and the Azores. For the purpose of this study, Alentejo and Algarve were analyzed as one region and Madeira and the Azores were analyzed as one region.

## RESULTS

All 41 ICMS directors answered the questionnaire.

The number of active ICM beds in ICMS is 842, and 77.3% of these are equipped for level 3 of care. There are 236 additional beds installed but not active in the ICMS, with 61.4% equipped for level 3 of care, and 130 of them not needing additional resources for the activation. Therefore. there are 8.04 active ICMS beds per 100,000 habitants (9.36 per 100,000 habitants above 18 years old) and 10.30 installed ICMS beds per 100,000 habitants (11.98 per 100,000 habitants above 18 years old). The ratio of beds per capita is heterogeneous in the different regions of Portugal. The ratio of active beds is higher in Lisbon and the Tagus River Region and in the Autonomic Regions (Madeira and the Azores), with respectively 9.51 and 9.53 beds per 100,000 habitants, and lower in Northern and Centre Regions (7.0). Regarding installed beds, the highest ratio is observed in Alentejo and the Algarve Region and Lisbon and the Tagus River Region, with respectively 14.31 and 12.35 per 100,000 habitants, and lower ratios are found in the Centre and Northern Regions, with respectively 7.62 and 8.82 beds per 100,000 habitants. Table 1 presents data regarding number, ratio, and type of ICMS beds. The number of active beds per 100,000 habitants more than doubled between 2010 and 2022, increasing from 4.2 to 8.8. However, the ratio decreased in the last two years to 8.0 (Table 2). In fact, active beds decreased from 2022 to 2024 in all regions, except the Centre region (Table 2). The activation of the non-active installed beds activatable without additional resources would mean an increase to 9.3 beds per 100,000 habitants (Table 1).

**Table 1:** Number, ratio, and type of Intensive Care Medicine Services beds by Portuguese health regions and nationally (Portugal)

	North	Centre	LTR region	Alentejo & Algarve	Madeira & Azores	Portugal
Number of NHS ICMS	12	7	13	5	4	41
Number of Level 3 active ICMS beds	224	94	238	54	41	651
Number of Level 2 active ICMS beds	53	41	61	30	6	191
Total number of active ICMS beds	277	135	299	84	47	842
Number of active ICMS beds per 100,000 habitants	7.00	7.00	9.51	8.90	9.53	8.04
Number of active ICMS beds per 100,000 adult habitants						9.36
Active ICMS beds/Active acute care hospital beds (%)	5.6	4.1	6.3	4.9	3.9	5.6
Number of Level 3 activatable beds without additional human resources	14	2	21	17	8	62
Number of Level 2 activatable beds without additional human resources	26	2	27	13	0	68
Total number of activatable beds without additional human resources	40	4	48	30	8	130
Number of active or activatable ICMS beds	317	139	347	114	55	972
Number of active or activatable ICMS beds per 100,000 habitants	8.01	7.20	11.04	12.08	11.15	9.29
Number of active or activatable ICMS beds per 100,000 adult habitants						10.8
Number of Level 3 ICMS installed beds that are non-active and non-activatable beds without additional human resources	31	8	27	13	4	83
Number of Level 2 ICMS installed beds that are non-active and non-activatable without additional human resources	1	0	14	8	0	23
Total number of ICMS installed beds that are non-active and non-activatable without additional human resources	32	8	41	21	4	106
Total number of ICMS installed beds per 100,000 habitants	8.82	7.62	12.35	14.31	11.96	10.30
Total number of ICMS installed beds per 100,000 adult habitants						11.98

LTR - Lisbon and Tagus River; NHS - National Health Service; ICMS - Intensive Care Medicine Services.

**Table 2:** Evolution of the number of active Intensive Care Medicine Services beds per 100,000 habitants from 2010 to 2024, using available data, in Portugal and in each the Portuguese health regions

Portuguese health regions	2010	2016	2019	2020	2022	2024
North		6.7		6.4	8.2	7.0
Centre		4.4		4.7	4.7	7.0
Lisbon and Tagus River Region		6.9		7.2	8.9	9.5
Alentejo & Algarve		6.9		7.3	9.0	8.9
Madeira & Azores						9.5
Portugal	4.2	6.0	6.4	6.5	8.8	8.0

Ninety-five percent of ICMS perform integrated management of patients in level 2 and level 3 of care and 76% of the ICMS state they practice plasticity of bed capacity (Table 3). Only 15% organize pathology dedicated cohorts, mostly neurocritical, and 61% have formal collaborative protocols with other services in the hospital. In 34% of the hospitals with ICMS, there are other level 2 or level 3 units, apart from coronary and stroke units, not included in the ICMS. The occupancy rate is equal to or above 85%, above 75%, and above 70% in 37%, 49%, and 80% of the ICMS, respectively. The majority – 87% – of the ICMS with occupancy rate equal to or above 85% have installed non-active beds. More than half of the ICMS directors – 51% – state that there are patients in the wards of their institution that would benefit from ICU admission, and this seems to be more frequent in the northern region. This perception occurs in 38% of the institutions with ICMS with occupancy rate below 80% (Table 3).

**Table 3:** Intensive Care Medicine Services occupancy rates and management of bed availability by Portuguese health regions and nationally (Portugal)

		North n = 12	Centre n = 7	LTR region n = 13	Alentejo & Algarve n = 5	Madeira & Azores n = 4	Portugal n = 41
Proportion of ICMS that practice plasticity and adaptation of the number of active beds		9/12	5/7	11/13	3/5	3/4	31/41
Proportion of ICMS that practice integrated management of level 2 and level 3 patients		10/12	7/7	13/13	5/5	4/4	39/41
Proportion of ICMS with organization of pathology dedicated cohorts or ICUs		2/12	0/7	4/13	0/5	0/4	6/41
Proportion of hospitals with other level 3 or level 2 ICUs (apart from stroke and coronary units) not included in the ICMS		4/12	3/7	4/13	3/5	0/4	14/41
Proportion of ICMS with collaborative protocols with other hospital services		6/12	3/7	9/13	3/5	4/4	25/41
Number of ICMS with last 12 months' occupancy rate							
	≥ 80%	7	3	9	0	1	20
	70 - 80%	3	3	3	4	0	13
	< 70%	2	1	1	1	3	8
Number of ICMS with last 12 months occupancy rate							
	≥ 85%	6	2	6	1	0	15
Proportion of ICMS with installed non-active beds, with occupancy rate above 85%		5/6	1/2	6/6	1/1	0/0	13/15
Proportion of ICMS directors with perception of the existence of patients in the ward that would benefit from ICU admission ("care gap")		10/12	4/7	6/13	1/5	0/4	21/41
Perception of "care gap" in hospitals with ICMS occupancy rate below 80%		3/12	1/7	3/13	1/5	0/4	8/21

LTR - Lisbon and Tagus River; ICMS - Intensive Care Medicine Services; ICU - intensive care unit.

The staff of the 41 ICMS includes 675 doctors with at least one specialty, 445 of them ICM specialists (66%). More than half of the others are in training to acquire this specialty. The percentage of intensivists among the ICMS staff is rather homogeneous throughout the country, although slightly lower in Algarve/Alentejo Region. In the morning shift, 93% of the ICMS have a ratio of senior doctors to beds not worse than 1/5 and none has a ratio worse than 1/7. At night, only 7% had ratios worse than 1/10. Medical resources consumption seems to be lower in the morning shift in North and Centre regions and at night in North and Alentejo/Algarve Regions (Table 4). The number of hours for non-clinical tasks is extremely heterogeneous, varying from 0 to 704 hours per month, and the motivation of the staff is perceived by the director as good or excellent in 70% of the ICMS. There is no rehabilitation nursing in 27% of the ICMS, and there is support for 12 hours per day, including weekends, in only 22% of them. ICMS have the support and participation of physiotherapists, psychologists, and social service professionals in 100%, 73%, and 78% of the cases, respectively (Table 4).

**Table 4:** Human resources in Intensive Care Medicine Services by Portuguese health regions and nationally (Portugal)

		North n = 12	Centre n = 7	LTR region n = 13	Alentejo & Algarve n = 5	Madeira & Azores n = 4	Portugal n = 41
Number of certified intensivists on ICMS staff		170	53	167	32	23	445
Number of MD specialists (any specialty) on ICMS staff		253	78	255	55	34	675
Percentage of intensivists among the ICMS medical staff %		67.2	67.9	65.5	58.2	67.6	65.9
Intensivists on ICMS staff per 100,000 habitants		4.29	2.75	5.31	3.39	4.66	4.25
Intensivists on ICMS staff per 100,000 adult habitants							4.94
ICMS medical staff per 100,000 habitants		6.39	4.04	8.11	5.83	6.89	6.45
ICMS medical staff per 100,000 adult habitants							7.50
Number of ICMS with the ratio of specialist MD per active bed in the morning shift							
	≤ 1/3	3	1	5	3	2	14
	> 1/3 and ≤ 1/5	8	4	8	2	2	24
	> 1/5 and ≤ 1/7	1	2	0	0	0	3
Number of ICMS with the ratio of specialist MD per active bed in the night shift							
	≤ 1/7	2	3	11	1	3	20
	>1/7 and ≤ 1/10	9	3	1	4	1	18
	> 1/10 and ≤ 1/12	1	0	1	0	0	2
	> 1/12	0	1	0	0	0	1
Number of hours for non-clinical tasks per month		2 - 563	5 - 300	0 - 704	0 - 152	0 - 10	0 - 704
Rehabilitation nursing coverage							
	12 hours per day every day (84 hours per week)	3	2	2	1	1	9
	Between 35 and 84 hours per week	6	1	4	1	0	12
Only in the morning on weekdays							
	None	2	3	2	1	1	9
Number of ICMS with a ratio of rehabilitation nurses per ICMS bed of at least 1/8 in the periods covered		0	1	3	2	1	7
Proportion of ICMS with support of physiotherapists		12/12	7/7	13/13	5/5	4/4	41/41
Proportion of ICMS with support of psychologist		9/12	4/7	10/13	4/5	3/4	30/41
Number of ICMS with support of social services worker		9/12	4/7	11/13	4/5	4/4	32/41
Number of ICMS with a motivational state classified by its director as							
	Excellent	2	2	2	1	1	8
	Good	4	3	7	4	2	20
	Intermediate	5	2	3	0	0	0
	Low	1	0	1	0	0	2

LTR - Lisbon and Tagus River; ICMS - Intensive Care Medicine Service; MD - medical doctor.

Regarding outreach (Table 5), including all the critically ill patient journey activities described above, 59% of the ICMS are responsible for the resuscitation room of the emergency department, 37% cover it with intensivists 24/7, 85% manage and operate the intra-hospital emergency team (IHET), and 75% perform intra-hospital and outpatient follow-up clinics. Outreach activities are much more commonly implemented in ICMS of the Northern Region, where 92% are responsible for the resuscitation area of the emergency department, 100% operate the IHET, and 92% operate follow-up clinics.

**Table 5:** Outreach activities of the Intensive Care Medicine Services by Portuguese health regions and nationally (Portugal)

	North n = 12	Centre n = 7	LTR region n = 13	Alentejo & Algarve n = 5	Madeira & Azores n = 4	Portugal n = 41
Number of ICMS that perform on the intra-hospital emergency team	12	5	11	3	4	35
Number of ICMS that manage the intra-hospital emergency team	12	5	11	3	2	33
Number of ICMS that manage the resuscitation room of the emergency department	11	4	7	1	1	24
Number of ICMS that provide at least one intensivist for the resuscitation room of the emergency department around the clock everyday	8	1	3	1	2	15
Number of ICMS that perform intra-hospital follow-up consultation	11	5	10	3	3	32
Number of ICMS that perform outpatient follow-up	11	3	10	5	3	32

LTR - Lisbon and Tagus River; ICMS - Intensive Care Medicine Services.

Regarding the referral network, 86% of the ICMS answered that they comply with the regulations of the Intensive Care Medicine Referral Network Document 2020. However, 88% of the services reported the existence of at least one constraint to patient referral. Moreover, 66% of all the ICMS stated that there were significant constraints in the secondary transport of the critically ill patient. Only 22% of all services reported lack of bed availability as one of the constraints, and 27% stated that there are difficulties in communications and lack of coordination and refereeing in cases of referral disagreements between centers.

## DISCUSSION

Intensive care unit bed capacity varies widely across countries. Low- and middle-income countries have significantly fewer ICU beds than high-income nations. For example, while Uganda and Bangladesh, respectively, have 0.1 and 0.8 adult critical care beds per 100,000 population, Taiwan and the United States have 28.5 and 27.0, respectively.^(12)^ The mean value for Organisation for Economic Co-operation and Development (OECD) countries in 2020 was 15.9.^(13)^ Our study shows that the number of ICMS beds in Portugal has markedly increased in the last 14 years, although the number of active beds has slightly decreased in the last two years. Portugal entered the pandemic with a low number of critical care beds, with just 6.4 critical care beds per 100,000 people, and during the pandemic period there was an increment, allowing a sustained enlargement of ICMS capacity. Currently, there are 9.36 active beds per 100,000 habitants above 18 years old, 77.3% of them equipped for level 3 of care, and 11.98 installed beds per 100,000 habitants above 18 years old. Only the activation of all the installed beds would attain the goal proposed for 2020 by the Referral Network Document 2016^(9)^ – 12.0 ICMS beds for 100,000 habitants – and this would still be below the averages for OECD and EU nations.

The baseline occupancy rate, using as denominator the number of active beds, is equal to or above 85% and above 75% in 37% and 49% of the ICMS, respectively. Moreover, 51% of the ICMS directors state that in their institution there are patients in the ward that would benefit from ICU admission, and this seems to be more frequent in the institutions in which the ICMS occupancy rate is higher. There are indications that although setting a uniform target figure for all ICUs would be problematic, as there are a range of factors both at the unit and the hospital level that impact occupancy levels, optimal ICU occupancy rates are around 70 - 75%,^(14)^ except in larger intensive care departments, in which 80 - 85% occupancy seems admissible. Therefore, it seems wise and adequate to activate all installed beds, providing the human resources needed for the purpose, especially as more than half of the non-active beds could be activated with no additional human resources, which would certainly reduce the gap of care, intensive care bed unavailability, and staff burnout. These currently inactive beds should be activated and viewed as baseline capacity and not as surge capacity. This is more needed in certain areas of the country, as the ratio of beds per capita is heterogeneous in the different regions of Portugal, being lower in the North and Centre regions. The resolution of this heterogeneity is very important, namely in terms of preparedness for surge periods. The COVID-19 pandemic underscored the importance of developing a robust, granular, and constantly updated understanding of the availability of intensive care beds, at different levels of geographic scale, and of incorporating this information into routine public health strategies and healthcare systems administration and planning.^(15)^ While it may be tempting to call for even more ICU beds, governments, policy makers, and the intensive care community must balance this, on one hand, with investments in more basic healthcare,^(16)^ and on the other hand, with the implementation of level 1 units in medical and surgical departments with telemetric communication with ICMS, using a broader, integrated, holistic pan-hospital view of the mission of ICMS through outreach provision of care.^(17)^

Although more than three-quarters of the beds are equipped for level 3 of care and most ICMS practice integrated management of level 3 and level 2 of care, allowing the attainment of a high degree of plasticity of bed management and of continuity of care, there is huge room for improvement in terms of the scope of their mission, namely in terms of outreach activities. In fact, most ICMS perform and manage intra-hospital emergency teams and follow-up clinics, but only 59% of the ICMS are responsible for the resuscitation room of the emergency department and only 37% cover it with intensivists 24/7. Critical disease is a continuum that clearly begins before ICU admission and, in survivors, extends far beyond ICU discharge. There is strong evidence of the impact of early recognition of the critical phase of diseases. Several studies demonstrate that patients show signs of deterioration in the 12 hours before intra-hospital cardiac arrest^(18)^ and that the implementation of a rapid response system, namely through an intra-hospital emergency team, reduces the incidence of intra-hospital cardiac arrest and unexpected death.^(19,20)^ The presence of an intensivist or intensive care team in the resuscitation room of the emergency department (ED), where the most severely ill patients are admitted, allows quick and adequate stratification of level of care, earlier stabilization, reduction of level 3 of care admissions and organ support, and mortality reduction.^(21)^ The presence of the intensivist in this early phase may also contribute to the reduction of the time of critically ill patients spent in the ED, and it is widely known that time in the ED is, in most studies, significantly and independently associated with longer length of hospital stay and higher mortality.^(22-25)^ It is probable that factors related to the organization of the ED, namely access to intensive care assessment in the ED or critical care competencies of the ED team, impact the effect of time in the ED before ICMS admission on outcome. However, this is yet to be proven and represents an interesting topic of future research. Moreover, the activity of an intensive care team in the ER and in the intra-hospital emergency team increases patient safety, reduces unexpected events, and improves outcomes at a hospital scale, as it also reduces no-value and low-value care.^(26)^ There are two main categories of low-value critical care: allocation of ICU beds to patients who will not benefit from it over admission to a ward and provision of excessive critical care resources to patients who appropriately gain entry to the ICU. While both are important, avoiding low-value ICU admissions portends generally greater cost reductions than avoiding waste from low-value services delivered to patients in the ICU.^(27)^ An adequate assessment of the value of critical care for a specific clinical situation significantly gains from the reasoning and decisions of an intensivist. Of course, not admitting to the ICU implies the participation of the intensive care team with other specialties, patients, and families in the design of the patient´s care plan. ICMS outreach activity is, in our opinion, a pre-requisite of a high-value ICU, conferring the following benefits: identifying critical patients earlier, selecting patients who will benefit from critical care services, and maximizing the rehabilitation process. Attaining the goal of all Portuguese ICMS performing all outreach activities, and acting in a full-mission mode, should be a healthcare priority.

Regarding ICMS staffing, the number of intensivists has significantly increased in the last ten years, but one-third of the medical staff is still composed of non-intensivists. This situation will probably be rectified in the coming years, as the pipeline of intensivists is still increasing, with an expected transitory overlap of specialists coming from residency/primary specialty and from supraspecialty certification. Seventy percent of the medical staff are perceived as possessing good or excellent motivation by the ICMS director. We did not investigate factors associated with motivation levels, but this would be a relevant area for future research. Several reviews have demonstrated the association between nurse staffing and clinical outcome, confirming that a ratio not worse than 1 nurse to 2 patients is a determinant of increased safety and better outcome.^(28-30)^ Although Portugal complies with this goal in terms of active beds, the number of nurses is clearly a reason for the existence of inactive beds. However, the main quality gap in terms of human resources lies in the absence of rehabilitation nurses covering 12 hours daily in 51% of ICMS, and their total absence in 27% of the ICMS. Early rehabilitation is an important determinant of good outcome and better quality of life for ICU survivors.^(31-33)^ A recently published study showed that intensive care units belonging to the cluster with full-time intensivists, dedicated pharmacists, and higher levels of nurse autonomy had the best outcomes, emphasizing the impact of ICMS structure on outcomes.^(34)^ Adequate critical care staffing is core to safety, outcomes, and patient experience. It is also vital for staff retention and motivation and for quality sustainability of ICMS.^(35)^

Regarding the Portuguese Intensive Care Referral Network, the guidelines published by the Portuguese Ministry of Health are generally complied with. However, two-thirds of the ICMS directors state that there are significant constraints with respect to the secondary transport of critically ill patients, causing transfer delays. In fact, due to the tertiarization and regionalization of care, namely of critical care, secondary transport is becoming more frequent.^(36)^ Swift stabilization and prompt referral to centers able to match care given to care needed impact outcomes.^(37)^ Secondary transport is an essential link of this referral system and, simultaneously, a potentially dangerous event. In Portugal, the secondary transport is based on an *ad-hoc* system, meaning that dedicated transport teams in the hospitals are an exception and that the nomination of the team is performed just before the transport on an as available basis. The availability of an ambulance is often difficult and constrained by time, and ambulance equipment is sometimes sub-optimal. This *ad-hoc* and dysfunctional system determines a heterogeneous non-standardized level of care, often inadequate to the patient's needs.^(38)^ Regarding the management of the referral network, communication, coordination, refereeing, and decision-making would benefit from the implementation of a National Information System that would be able to acquire and process constantly updated online information about bed availability and work burden.^(39)^

The main limitation of the study is that it is rooted in the narrative of ICU directors and not in direct institutional raw data. However, this is simultaneously a strength, as ICU directors are probably the best persons to inform and describe the actual picture of their own services. We also acknowledge that some of the questions evoke opinions and perceptions and not facts, but, again, this information originates from persons working professionally in the field, both as clinicians and health managers.

## CONCLUSION

The number of active intensive care medicine beds in Portugal has markedly increased in the last 12 years, but the beds/habitant ratio is still below the OECD average. The activation of all the installed beds would probably allow for the reduction of the hospital care gap perceived by many of the intensive care unit directors. There is significant geographic heterogeneity in the beds/habitant ratio and in the performance of outreach activities by Intensive Care Medicine Services. The number of intensivists is rapidly growing, but nursing staff should be augmented, especially rehabilitation nurses. The referral network is globally complied with, but the secondary transport of critical patients has much room for improvement and a constantly updated electronic information system can potentially aid in the day-to-day and strategic management of the Portuguese critical patient network.

## References

[1] Lassen HC (1954). The epidemic of poliomyelitis in Copenhagen, 1952. Proc R Soc Med.

[2] Vincent JL (2013). Critical care--where have we been and where are we going?. Crit Care.

[3] World Health Organization European Health Information Gateway. Number of acute care hospital beds.

[4] Halpern NA, Goldman DA, Tan KS, Pastores SM (2016). Trends in critical care beds and use among population groups and Medicare and Medicaid beneficiaries in the United States: 2000-2010. Crit Care Med.

[5] Ewbank L, Thompson J, McKenna H, Anandaciva S, Ward D NHS hospital bed numbers: past, present, future.

[6] Vincent JL, Creteur J (2019). Critical care medicine in 2050: less invasive, more connected, and personalized. J Thor Dis.

[7] Vincent JL (2019). The continuum of critical care. Crit Care.

[8] Ostermann M, Vincent JL (2023). ICU without borders. Crit Care.

[9] Paiva JA, Fernandes F, Granja C, Esteves F, Ribeiro J, Nóbrega JJ, Vaz J, Coutinho P (2016). Rede de Referenciação de Medicina Intensiva.

[10] Nuñez D, Gouveia J, Almeida-Sousa JP, Paiva JÁ, Bento L, Moreira P, Araújo R (2020). Proposta de Rede Nacional de Especialidade Hospitalar e de Referenciação – Medicina Intensiva.

[11] Instituto Nacional de Estatística (INE) Statistics Portugal.

[12] Phua J, Faruq MO, Kulkarni AP, Redjeki IS, Detleuxay K, Mendsaikhan N, Sann KK, Shrestha BR, Hashmi M, Palo JEM, Haniffa R, Wang C, Hashemian SMR, Konkayev A, Mat Nor MB, Patjanasoontorn B, Nafees KMK, Ling L, Nishimura M, Al Bahrani MJ, Arabi YM, Lim CM, Fang WF, Asian Analysis of Bed Capacity in Critical Care (ABC) Study Investigators, and the Asian Critical Care Clinical Trials Group (2020). Critical Care Bed Capacity in Asian Countries and Regions. Crit Care Med.

[13] British Medical Association (BMA) NHS hospital beds data analysis.

[14] Tierney LT, Conroy KM (2014). Optimal occupancy in the ICU: a literature review. Aust Crit Care.

[15] Kempker JA, Stearns E, Peterson EN, Walter LA (2023). U.S. adult critical care beds per capita: a 2021 county-level cross-sectional study. Crit Care Explor.

[16] Schultz MJ, Dunser MW, Dondorp AM, Adhikari NK, Iyer S, Kwizera A, Lubell Y, Papali A, Pisani L, Riviello BD, Angus DC, Azevedo LC, Baker T, Diaz JV, Festic E, Haniffa R, Jawa R, Jacob ST, Kissoon N, Lodha R, Martin-Loeches I, Lundeg G, Misango D, Mer M, Mohanty S, Murthy S, Musa N, Nakibuuka J, Serpa A, Nguyen Thi Hoang M, Nguyen Thien B, Pattnaik R, Phua J, Preller J, Povoa P, Ranjit S, Talmor D, Thevanayagam J, Thwaites CL, Global Intensive Care Working Group of the European Society of Intensive Care Medicine (2017). Current challenges in the management of sepsis in ICUs in resource-poor settings and suggestions for the future. Intensive Care Med.

[17] Phua J, Hashmi M, Hanifa R (2020). ICU beds: less is more? Not sure. Intensive Care Med.

[18] Churpek MM, Yuen TC, Winslow C, Robicsek AA, Meltzer DO, Gibbons RD (2014). Multicenter development and validation of a risk stratification tool for ward patients. Am J Respir Crit Care Med.

[19] Winters BD, Weaver SJ, Pfoh ER, Yang T, Pham JC, Dy SM (2013). Rapid-response systems as a patient safety strategy: a systematic review. Ann Intern Med.

[20] Hillman K, Chen J, Cretikos M, Bellomo R, Brown D, Doig G, Finfer S, Flabouris A, MERIT study investigators (2005). Introduction of the medical emergency team (MET) system: a cluster-randomised controlled trial. Lancet.

[21] Nguyen HB, Rivers EP, Havstad S, Knoblich B, Ressler JA, Muzzin AM (2000). Critical care in emergency department: a physiologic assessment and outcome evaluation. Acad Emerg Med.

[22] Weingart SD, Sherwin RL, Emlet LL, Tawil I, Mayglothling J, Rittenberger JC (2013). ED intensivists and ED intensive care units. Am J Emerg Med.

[23] Wessman BT, Mohr NM (2022). Emergency department ICU´s add value. Crit Care Med.

[24] Jayaprakash N, Pflaum-Carlson J, Gardner-Gray J, Hurst G, Coba V, Kinni H (2020). Critical care delivery solutions in the emergency department: evolving models in caring for ICU´s boarders. Ann Emerg Med.

[25] Jones S, Moulton C, Swift S, Molyneux P, Black S, Mason N (2022). Association between delays to patient admission from the emergency department and all-cause 30-day mortality. Emerg Med J.

[26] Gawande AA, Colla CH, Halpern SD, Landon BE (2014). Avoiding low-value care. N Engl J Med.

[27] Anesi GL, Wagner J, Halpern SD (2017). Intensive Care Medicine in 2050: toward an intensive care unit without waste. Intensive Care Med.

[28] Penoyer DA (2010). Nurse staffing and patient outcomes in critical care: a concise review. Crit Care Med.

[29] Needleman J, Buerhaus P, Pankratz VS, Leibson CL, Stevens SR, Harris M (2011). Nurse staffing and inpatient hospital mortality. N Engl J Med.

[30] West E, Barron DN, Harrison D, Rafferty AM, Rowan K, Sanderson C (2014). Nurse staffing, medical staffing and mortality in intensive care: an observational study. Int J Nurs Stud.

[31] Knutsen K, Solbakken R, Normann B (2024). The diverse invitations to participate in early rehabilitation – A qualitative study of nurse-patient interactions in the intensive care unit. Intensive Crit Care Nurs.

[32] Hodgson CL, Schaller SJ, Nydahl P, Timenetsky KT, Needham DM (2021). Ten strategies to optimize early mobilization and rehabilitation in intensive care. Crit Care.

[33] Murooka Y, Sasabuchi Y, Takazawa T, Matsui H, Yasunaga H, Saito S (2023). Long-term prognosis following early rehabilitation in the ICU: a retrospective cohort study. Crit Care Med.

[34] Soares M, Salluh JI, Zampien FG, Bozza FA, Kurtz PM (2024). A decade of the ORCHESTRA study: organizational characteristics, patient outcomes, performance and efficiency in critical care. Crit Care Sci.

[35] Intensive Care Society (2022). Joint Position Statement on Critical Care Staffing Standards. The Intensive Care Society, Faculty Intensive Care Medicine and UK Critical Care Nursing Alliance.

[36] Droogh JM, Smit M, Hut J, de Vos R, Ligtenberg JJ, Zijlstra JG (2012). Inter-hospital transport of critically ill patients; expect surprises. Crit Care.

[37] Cardoso LT, Grion CM, Matsuo T, Anami EH, Kauss IA, Seko L (2011). Impact of delayed admission to intensive care units on mortality of critically ill patients: a cohort study. Crit Care.

[38] Grier S, Brant G, Gould TH, von Vopelius-Feldt J, Thompson J (2020). Critical care transfer in an English critical care network: analysis of 1124 transfers delivered by an ad-hoc system. J Intensive Care Soc.

[39] McGovern M, Quinlan M, Doyle G, Moore G, Geiger S (2018). Implementing a National Electronic Referral Program: Qualitative Study. JMIR Med Inform.

